# The feasibility of low-concentration contrast and low tube voltage in computed tomography perfusion imaging: an animal study

**DOI:** 10.1042/BSR20170977

**Published:** 2018-01-10

**Authors:** Yuning Pan, Aiqin Song, Shizhong Bu, Zhaoqian Chen, Qiuli Huang, Aijing Li

**Affiliations:** 1 Department of Radiology, Ningbo First Hospital, Ningbo, China; 2 Department of Radiology, Jimo Traditional Chinese Medicine Hospital, Qingdao, China; 3 Diabetes Research Center, School of Medicine, Ningbo University, Ningbo, China; 4 Department of Radiology, Ningbo No.2 Hospital, Ningbo, China

**Keywords:** AIDR-3D reconstruction, computed tomography, liver, low-concentration contrast, low tube voltage, perfusion imaging

## Abstract

Aim**:** To investigate the feasibility of low-concentration contrast (270 mg/ml) together with low tube voltage (80 kV) and adaptive iterative dose reduction (AIDR)-3D reconstruction in liver computed tomography (CT) perfusion imaging.

Method**:** A total of 15 healthy New Zealand rabbits received two CT scans each. The first scan (control) was acquired at 100 kV and 100 mA with iopromide (370 mg/ml), while the second scan (experimental) was acquired at 80 kV and 100 mA with iodixanol (270 mg/ml) 24 h after the first scan. The obtained images were reconstructed with filtered back projection (FBP) and AIDR-3D in the control and experimental groups respectively. The perfusion parameters (hepatic artery perfusion [HAP], portal vein perfusion [PVP], hepatic perfusion index [HPI], and total liver perfusion [TLP]) and image quality (image quality score, average CT value of abdomen aorta, signal-to-noise ratio [SNR], contrast-to-noise ratio [CNR], and figure of merit [FOM]) were compared using a paired *t*-test or Mann–Whitney *U* test between the two groups, when appropriate. The effective radiation dose and iodine intake were also recorded and compared.

Results**:** With the exception of the FOM criteria, the image quality and perfusion parameters were not significantly different between the two groups. The effective radiation dose and iodine intake were 38.79% and 27.03% lower respectively, in the experimental group.

Conclusion**:** Low-concentration contrast (iodixanol, 270 mg/ml) together with low tube voltage (80 kV) and AIDR-3D reconstruction help to reduce radiation dose and iodine intake without compromising perfusion parameters and image quality in liver CT perfusion imaging.

## Introduction

Computed tomography perfusion imaging (CTPI) is a fast, accurate, and noninvasive functional imaging technique that reflects hemodynamic changes in the microcirculation of organs and tissues. This technique has been widely employed in the quantitative analysis of acute ischemic stroke in the brain [1–3]. As post-processing strategies and software have developed, the application of CTPI has expanded to more settings, including the assessment of hepatic and pancreatic lesions [4–10]. Specifically, liver CTPI has been shown to provide valuable information for the diagnosis and prognosis of liver diseases such as hepatic cirrhosis, hepatocellular carcinoma, and cancer metastasis to the liver [7–14].

To improve the image quality associated with liver CTPI, two methods are generally applied: increasing contrast agent concentration and enhancing X-ray energy. However, both strategies are inappropriate in terms of patient safety. On one hand, higher concentrations of iodine contrast agent may lead to an increased burden in the patients’ hematological and urinary systems [15]. On the other hand, higher X-ray energy is also not practical due to radiation dose limitations, especially since the liver is radiation-sensitive, and liver CTPI requires multiple acquisitions of the same region. Cohnen and colleagues showed that the radiation dose associated with liver CTPI is 6.7 times higher than a regular abdominal CT [16]. As a result, obtaining precise diagnostic liver CTPI images with minimal radiation and iodine contrast doses is clinically important [9,16].

With 320-slice CT scans, it is possible to perform dynamic imaging of the entire liver, given that the facility provides a 16 cm axial field of view (FOV). This also helps reduce radiation dose without compromising image quality [13]. Lower tube voltage (80 kV) results in increased attenuation from the contrast agent, which in turn leads to a higher CT value of the vessels when compared with those used in regular body perfusion imaging (100 kV). This allows lowering of the contrast agent concentration, and therefore reduces side effects associated with the contrast agent. With the traditional reconstruction technique (filtered back projection [FBP]), radiation-dose reduction causes an increase in the image noise. However, iterative reconstruction (IR) techniques have been shown to reduce image noise and radiation dose and improve image quality [17,18]. Low tube voltage together with low-concentration contrast agent have been increasingly used in recent years, but most studies have focused on coronary arteries, head and neck, and pulmonary arteries [17,19–21]. The application toward body perfusion imaging has not been well studied.

The purpose of the present study was to investigate the feasibility of isotonic low-concentration contrast agent together with low tube voltage and AIDR-3D in liver CTPI.

## Methods

### Animal model

The present study was approved by the Institutional Animal Care and Use Committee (IACUC) at Ningbo First Hospital, Ningbo, China. Fifteen healthy New Zealand white rabbits (weight: 2.3 ± 0.3 kg; age: 12 months; seven males and eight females) were provided by the Animal Experiment Center of Ningbo University (Production License Number: SYXK [Zhe] 2013-0191) and used in the present study.

### Animal preparation

The rabbits were fed green vegetables 1 day before each CT scan to avoid high-density artifacts in the intestine. In addition, all rabbits were fasted for 6 h prior to the imaging studies.

### Image acquisition

The rabbits were fixed in the supine position on a self-made table and anesthetized with 3% pentobarbital (1 ml/kg) via a heparinized indwelling catheter in the marginal ear vein. Abdominal compression was applied with a secured belly band to reduce respiratory motion of the liver, thereby reducing motion artifacts. A 320-slice spiral CT (Aquilion One, Toshiba Medical, Japan) was used for imaging. The following dynamic volume scan mode was used: collimator width = 160 mm, FOV 150 mm × 150 mm, and slice thickness = 0.5 mm. Each rabbit was scanned twice; the first scan was set as the control group and the second scan was set as the experimental group. The first scan was acquired at 100 kV and 100 mA with iopromide (Ultravist 370 mg/ml, Bayer, Guangzhou, China), while the second scan was acquired at 80 kV and 100 mA with iodixanol (Visipaque 270 mg/ml, General Electric, Shanghai, China) 24 h after the first scan to eliminate any influence from the Ultravist.

The contrast agents were injected to the indwelling catheter with a dual chamber power injector (Stellant, Medrad, U.S.A.) at a dosage of 1.5 ml/kg and rate of 1.5 ml/s. After the injection, the catheter was washed with 10 ml of saline solution at the same rate. For the CTPI acquisitions, 23 volumes (each containing 320 slices) were acquired. Each of the 23 volumes took 0.5 s to scan, and the temporal sampling intervals were 2 s (volume 1–11), 3 s (volume 12–18), and 5 s (volume 19–23). The total acquisition time was 74.5 s.

### Perfusion parameters

The acquired images were reconstructed using FBP and 50% AIDR-3D in the control and experimental groups respectively. The raw data acquired from the dynamic CT were transferred to a commercial workstation (Aquilion One, Toshiba Medical, Otawara, Japan) after correcting for respiratory motion. The data were analyzed using special body perfusion software (Vitrea Fx6.2.3, Vital Images, Minnertoka, MN, U.S.A.). Perfusion parameters were calculated from the image volumes using a deconvolution algorithm [22]. The soft tissue threshold was chosen based on the recommended value by the software: 0–120 HU and a region of interest (ROI) of 50 mm^2^. The abdominal aorta and portal vein at the hepatic hilum level were identified as the input artery and input vein respectively. The ROIs were positioned at the hepatic parenchyma (segment IV) and the center of the splenic parenchyma [23], excluding blood vessels. Tissue perfusion was estimated on a segmental basis by calculating time-density curves (TDC) from the dynamic CT volumes.

The following perfusion maps were obtained: hepatic artery perfusion (HAP), portal vein perfusion (PVP), and hepatic perfusion index (HPI). ROIs were independently placed by two radiologists (Y.N.P. and A.Q.S. with 12 and 8 years of experience in abdominal radiology respectively). ROIs were located at the lateral sector of left lobe, medial sector of left lobe, and right lobe at the level of the main portal vein, avoiding major vessels and the bile duct. Each measure was repeated three times, and the average was used for further analysis. Total liver perfusion (TLP), equal to the sum of HAP and PVP, was also evaluated.

### Image quality

#### Subjective image quality evaluation

Two radiologists (Y.N.P. and A.J.L. with 12 and 9 years of experience in abdominal radiology respectively) blinded to the scanning parameters independently reviewed the acquired data at the workstation. The quality of the images was evaluated used a 5-point scoring scale: [24] 5 = excellent; 4 = good; 3 = fair; 2 = poor; and 1 = unacceptable. An image was considered clinically acceptable if it scored 3 points or higher.

#### Objective image quality evaluation

Objective image quality evaluation was performed using the peak of the TDC on the original axial images. The CT value and standard deviation (SD) of the abdominal aorta (the central 70–80%) and musculus erector spinae (a circular ROI with an area of approximately 50 mm^2^) at the level of the hepatic hilum were measured. All measurements were repeated three times in adjacent slices, avoiding large blood vessels and artifacts, and the mean values were used for further analysis. The following CT parameters were subsequently evaluated: SNR (average CT value of abdominal aorta/SD of abdominal aorta); CNR ((average CT value of abdominal aorta − average CT value of musculus erector spinae)/SD of musculus erector spinae). The figure of merit (FOM) was defined as FOM = CNR^2^/effective dose.

### Estimation of radiation dose and iodine intake

The effective radiation dose was assessed with the volume CT dose index (CTDIvol) and dose length product (DLP). The effective dose was defined as: DLP × conversion coefficient for the abdomen (*k* = 0.015 mSv mGy^−1^ cm^−1^).

Iodine intake (total iodine and flow rate) was evaluated using the following formula: total iodine (*g*) = concentration of contrast agent (mg/ml) × volume of contrast agent (ml)/1000.

### Statistical analysis

Descriptive statistics were used to depict means and SDs. A paired *t*-test was applied to evaluate differences in the experimental and control groups regarding the following parameters: perfusion (HAP, PVP, HPI, and TLP), image quality (SNR, CNR), effective radiation dose, and iodine intake.

A Mann–Whitney *U* test was employed to assess differences in image quality as measured by the 5-point scale. The inter-rater reliability between two radiologists was assessed with Cohen’s kappa coefficient, which categorizes values as having poor agreement (<0.4), fair agreement (<0.41–0.60), moderate agreement (<0.61–0.80), or excellent agreement (<0.81–1.0). All analyses were carried out using SPSS v19.0 software (SPSS Inc., Chicago, IL, U.S.A.). A *P* value of less than 0.05 was considered statistically significant.

## Results

### Perfusion parameters

As shown in Table 1, no differences were revealed in HAP, PVP, HPI, and TLP between the control and experimental groups.

**Table 1:** The difference of CT perfusion parameters between experimental and control groups

Parameters	Experimental group	Control group	*p*
HAP (ml/(100 g min))	33.76 ± 1.85	33.63 ± 2.01	0.65
PVP (ml/(100 g min))	94.90 ± 2.47	95.85 ± 3.28	0.39
HPI	0.26 ± 0.01	0.26 ± 0.02	0.46
TLP (ml/(100 g min))	128.67 ± 0.91	129.47 ± 0.78	0.45

Abbreviations: CT, computed tomography; HAP, hepatic artery perfusion; HPI, hepatic perfusion index; PVP, portal vein perfusion; TLP, total liver perfusion.

### Image quality

**Figure 1:**
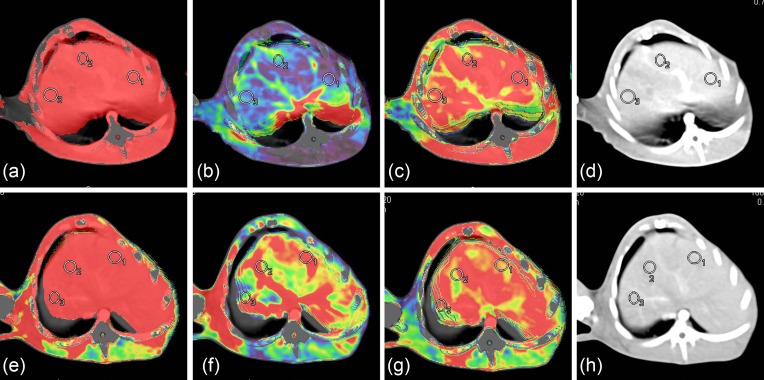
CT liver perfusion images in rabbit. CT perfusion images in control (**a**–**d**) and experimental (**e**–**h**) groups (ROIs are marked in circles). (a) and (e): hepatic artery perfusion images; (b) and (f): portal vein perfusion images; (c) and (g): hepatic perfusion index; (d) and (h): maximal intensity projection; CT: computed tomography; ROI: regions of interest.

The image quality was satisfactory using both methods (Figure 1). The average image quality scores were 4.0 ± 0.76 and 4.3 ± 0.62 in the control and experimental groups respectively. All acquired images were clinically acceptable and no differences were found between the two groups (*P*>0.05). The average CT values of the abdominal aorta in both groups were higher than 250 HU, with no significant differences identified (*P*>0.05, Table 2). The SNR and CNR were similar between the two groups (*P*>0.05, Table 2); however, the FOM in the experimental group was significantly higher than the control group (*P*<0.05, Table 2).

**Table 2:** The difference of SNR and CNR between experimental and control groups

Parameters	Experimental group	Control group	*p*
The CT value of abdomen aorta (HU)	305.74 ± 38.43	299.66 ± 24.64	0.59
SNR	21.30 ± 1.28	22.33 ± 1.61	0.14
CNR	28.98	30.10	0.06
FOM	44.67 ± 24.26	29.25 ± 13.03	0.00

Abbreviations: CNR, contrast-to-noise ratio; CT, computed tomography; FOM, figure of merit; SNR, signal-to-noise ratio.

The reading agreement between two radiologists was excellent (kappa = 0.81, *P*<0.05).

### Radiation dose and iodine intake

The effective radiation dose was 38.79% lower in the experimental group compared with the control group (19.85 vs. 32.43 mSv respectively). The injected doses of contrast agent were similar between the two groups; however, the iodine intake was 27.03% lower in the experimental group compared with the control group (0.97 g vs. 1.32 g, *P*=0.00).

## Discussion

Our data revealed that rabbits injected with iodixanol and scanned at 80 kV (experimental group) showed higher FOM, received a lower effective radiation dose, and experienced lower iodine intake compared with those injected with iopromide and scanned at 100 kV (control group). The results presented above were achieved without compromising image quality, and were characterized by similar image quality score, average CT values of the abdominal aorta, SNR, and CNR, as well as HAP, PVP, HPI, and TLP in both groups. This evidence suggests that low tube voltage together with a low-concentration contrast agent can potentially reduce the side effects of CT scans while maintaining equivalent image quality.

Previous research has shown that low-concentration contrast impaired enhancement of a blood vessel compared with the high-concentration equivalent in body perfusion image, even though the total contrast dose and flow rate were the same; this subsequently led to compromised image quality and inaccurate diagnoses [25,26]. The enhancement of a blood vessel can be achieved by increasing the flow rate of the contrast agent or by using a higher concentration of the contrast agent. However, a higher flow rate leads to an increased risk of contrast agent leakage, and increased concentration of the contrast (e.g. 400 mgI/ml) and could result in high viscosity, which contributes to difficult manipulation. Hence, low-concentration contrast is a better choice as it reduces the renal iodine burden for the patient as well as the risk of leaking. Even though enhancement would be decreased due to the low-concentration contrast, the low tube voltage compensates for the reduced CT value.

Ionization is another major drawback of the standard CT scan. Currently, several strategies have been applied to reduce radiation dose. Lowering the tube voltage—based on the principle that the X-ray penetration is dependent on the tube voltage and the exponential characteristics of the radiation dose—is one of the most commonly used methods. Compared with scans with lower voltage, the average energy of the photons is closer to the K-edge of iodine, and there is lower Compton scattering. Therefore, the CT value increases as the radiation dose decreases [27,28].

The above theories provide strong evidence supporting low-concentration contrast agents as an appropriate option. In our work, total iodine intake was 27.03% less in the experimental group, although the contrast dose and injection time were equivalent in the two groups. The reduction in contrast dose did not impair the image quality, since low voltage scanning served as an excellent compensator and the average CT value of abdominal aorta in the experimental group was above 250 HU, which was adequate for clinical diagnosis.

Results presented here provide further evidence to support the fact that low tube voltage scanning helps to increase the CT value in the artery. However, due to the lower X-ray photon energy, artifacts caused by beam hardening will increase. As a result, image quality will be significantly impacted. This disadvantage has limited the future application of low tube voltage scanning in the clinical setting.

AIDR-3D iterative reconstruction is a novel image reconstruction method recently applied in the clinical practice. Compared with traditional FBP, AIDR-3D is a hybrid algorithm combining reconstruction and noise reduction in an iterative fashion. The algorithm is capable of adapting to different scanning parameters and scanners by explicitly incorporating the statistical noise models and the scanner information into reconstruction of the projection data with the filter strength based on the relative noise level. Furthermore, anisotropic diffusion is used in the image domain with edge preservation for denoising to maintain edge structures while decreasing the noise level. These advantages have been proven in coronary, head, and neck CT angiography [28–30] and provide evidence to support the application of AIDR-3D in liver perfusion imaging.

Recent research compared reconstructed image quality using different AIDR strengths. It was found that a stronger level leads to a lower image noise level; however, increased blurring in the images would impact the diagnosibility, and noise reduction was insufficient with mild AIDR strength [28,31]. Hence, the standard level is generally recommended. Therefore, in the present study, we chose 50% AIDR for IR to reduce image noise and to enhance image quality.

The application of low tube voltage (80 kV) together with AIDR-3D reconstruction has achieved comparable image noise, SNR, CNR, and image quality scores when compared with the combination of regular voltage (100 kV) and FBP reconstruction, which was concordant with a previous publication [32]. Our data also showed a lower effective radiation dose in the experimental group (19.85 mSv) compared with that of the control group (32.43 mSv), which was more preferable than that reported by Zhang et al. [22]. This could be a result of volume acquisition and an improved acquisition protocol.

SNR, evaluated as the ratio of the CT value and SD in a given area, is valuable in assessing image quality. In perfusion imaging, a higher SNR represents distinguished density differences between the artery and other tissues. In this case, small vessels can be shown in detail, which results in increased perfusion quality. In our work, no differences were revealed in terms of the SNR and CNR of the abdominal aorta between the two groups, suggesting that low tube voltage together with low-concentration contrast and iterative reconstruction do not compromise CT perfusion imaging in a rabbit model. Similarly, Zheng et al. [21] reported that low-concentration contrast did not affect SNR in coronary CT angiography. The concept of FOM—which combines SNR, CNR, and radiation dose—has recently been more frequently applied in assessing the impact of radiation dose on image quality. An increased FOM represents better CT image quality. In our work, although the CNR did not decrease, the FOM increased significantly in the experimental group because of the decreased effective dose, thus indicating that low tube voltage can help to improve perfusion image quality.

There are some noteworthy advantages in the present study. First, the present study utilized 320-slice CTPI using dynamic volume acquisition with a 16 cm axial FOV without table movement. The high temporal resolution and spatial resolution ensured good image quality. Image registration was also effective at reducing motion artifact. Whole-liver CTPI enabled perfusion imaging of the entire organ in a single acquisition, and made ROI selection more flexible, thereby improving perfusion parameter estimation accuracy [6,33]. The present study used a deconvolution algorithm in liver, and TLP was found to be (129.47 ± 0.78) and (128.67 ± 0.91) ml/(100 g min) in the control group and experimental group respectively, which was similar to the results previously reported by Materne et al. [34] using microsphere experiments. HPI was found to be 0.26 ± 0.02 and 0.26 ± 0.01 in the control group and experimental group respectively, which was close to the normal ratio between the hepatic artery and portal vein (1:3–4), indicating that deconvolution helps to precisely evaluate the hemodynamics in the liver and hepatic perfusion status. Finally, the design of the present study, in that each experimental condition had a matched control condition, helped to reduce individual differences. Such a strategy could hardly be fulfilled during a human study. We conducted two separate scans at an interval of 24 h; this wash-out period helped to eliminate the impact of the first contrast agent, and therefore generated more accurate results.

A few limitations in our study are also worth noting. First, as a healthy rabbit model has a relatively small liver, its liver function could be significantly different from a pathological model, which could have led to more imaging artifacts, higher estimated perfusion, and limited generalizability. In addition, the physiological and histological characteristics of the human liver are different from that of the rabbit. Future research is necessary to investigate the response in human subjects and to optimize the combination of radiation and contrast doses in the clinical setting. Finally, the sample size used in the present study was relatively small. A larger trial and more complex study design will be beneficial toward identifying the application of the proposed strategy.

In summary, low-concentration contrast (iodixanol) together with low tube voltage (80 kV) and AIDR-3D reconstruction help to reduce radiation dose and iodine intake without compromising perfusion parameters and image quality in liver CT perfusion imaging in the healthy rabbit model.

## Abbreviations

AIDR: adaptive iterative dose reduction
CNR: contrast-to-noise ratio
CT: computed tomography
CTPI: computed tomography perfusion imaging
FBP: filtered back projection
FOM: figure of merit
FOV: field of view
HAP: hepatic artery perfusion
HPI: hepatic perfusion index
PVP: portal vein perfusion
ROI: region of interest
SNR: signal-to-noise ratio
TDC: time-density curves
TLP: total liver perfusion
